# CXCL9 inhibition does not ameliorate disease in murine models of both primary and secondary hemophagocytic lymphohistiocytosis

**DOI:** 10.1038/s41598-023-39601-9

**Published:** 2023-07-29

**Authors:** Tamir Diamond, Michelle Lau, Jeremy Morrissette, Niansheng Chu, Edward M. Behrens

**Affiliations:** 1 Division of Gastroenterology, Hepatology and Nutrition, The Children’s Hospital of Philadelphia, Philadelphia, PA USA; 2 Department of Pediatrics, University of Pennsylvania, Perelman School of Medicine, Philadelphia, PA USA; 3 Department of Immunology, University of Pennsylvania, Perelman School of Medicine, Philadelphia, PA USA; 4 Division of Rheumatology, The Children’s Hospital of Philadelphia, Philadelphia, PA USA

**Keywords:** Chemokines, Cytokines, Immunological disorders, Inflammation, Hepatology, Paediatric rheumatic diseases

## Abstract

Hemophagocytic Lymphohistiocytosis (HLH) is a group of disorders culminating in systemic inflammation and multi-organ failure with high incidence of hepatic dysfunction. Overproduction of IFN-γ is the main immunopathological driver in this disorder. Monokine induced by IFN-γ (CXCL9) serves as a biomarker for disease activity and response to treatment in this disorder. However, very little is understood about the actual functional role of CXCL9 in pathogenesis in HLH. In the current study, we sought to determine the role of CXCL9 in pathogenesis in murine models of both Familial HLH (*prf1*^*−/−*^*)* and Toll Like Receptor (TLR) 9 repeated stimulation induced Macrophage Activation Syndrome (MAS), a form of secondary HLH. FHL and MAS were induced in both CXCL9 genetically deficient mice (*cxcl9*^*−/−*^) and controls as well as using AMG487, a pharmacological antagonist of the CXCL9 receptor, CXCR3. Results showed that CXCL9 genetic deficiency did not improve disease parameters or hepatitis in both models. Consistent with genetic ablation of CXCL9, inhibition of its receptor, CXCR3, by AMG487 did not show any significant effects in the FHL model. Taken together, inhibition of CXCL9-CXCR3 interaction does not ameliorate HLH physiology in general, or hepatitis as a classical target organ of disease.

## Introduction

Hemophagocytic Lymphohistiocytosis (HLH) is a group of disorders that culminate in systemic inflammation affecting multiple organs and driving high rates of morbidity and mortality^1^. Causes of HLH are defined as primary when they arise from genetic mutations such as the perforin (*PRF1)* gene, which results in defects in cytolytic pathway components causing familial HLH (FHL). The syndrome is labelled secondary HLH if it occurs as a sequalae of another condition such as rheumatologic (i.e. Macrophage Activation Syndrome), malignancy, immunodeficiency, infection or inborn error of metabolism^2^. In FHL, disabled perforin mediated cytolysis results in the prolonged contact of CD8+ T-cells with their targets including antigen presenting cells (APC)^3^. The subsequent hyperstimulation of CD8+ T-cells and overproduction of IFN-γ by CD8+ T cells is the main immunopathological driver in this disorder both clinically and in experimental models^4–8^. One of the main manifestations of FHL and Macrophage Activation Syndrome (MAS) is hepatic dysfunction, which results in high morbidity and mortality and is the cause of 3% of cases of pediatric acute liver failure (PALF) predominantly in the neonatal period^9–12^. In humans, a liver specific IFN-γ response has been described based on gene transcription from biopsies^13^.

Extensive experimental and observational literature describe the importance of chemokine receptor 3 (CXCR3)-associated ligands: monokine induced by IFN-γ (MIG/CXCL9), IFN-γ-inducible protein 10 (IP-10/CXCL10) and IFN-inducible T cell α chemoattractant (I-TAC/CXCL11) in T-cell mediated hepatic immunopathology^13–20^. Production of these chemokines, predominantly CXCL9, is induced by IFN-γ as part of the inflammatory response and results in further recruitment of CXCR3+ T cells to affected tissue^15,18^. The liver is particularly affected in FHL with many patients developing significant hepatic dysfunction^12,21–23^. The liver additionally presents a unique opportunity for IFN-γ driven pathology, as hepatocytes express an IFN-γ receptor (IFNγ-R) and therefore allow for CXCL9 production^24,25^. In addition, CXCL9 is localized to hepatic sinusoids at baseline and during acute inflammation. This facilitates further CXCR3+ T cell recruitment during inflammation independently from leukocyte production^14,24,26,27^.

While CXCL9 has been established as an important biomarker for IFN-γ activity in Familial HLH (FHL) and secondary HLH, very little is understood about the location, kinetics, and quality of the tissue responses to IFN-γ and the functional role of CXCL9 in pathogenesis in HLH^20,28^. Prencipe et al. described that hepatic CXCL9 is upregulated transcriptionally in humans with secondary HLH/MAS^13^. In addition, our group has shown that hepatic transcription of this chemokine is primarily dependent on the hepatic IFN-γ response^25^. While following CXCL9 levels is common practice as a diagnostic and a measurement of disease activity in HLH, understanding the biological role of CXCL9 in pathogenesis of HLH remains a critical gap in knowledge.

In the current study, we sought to determine the role of CXCL9 in pathogenesis in murine models of both FHL (*prf1*^*−/−*^*)* and Toll Like Receptor (TLR) 9 repeated stimulation induced MAS^4,29^. The FHL murine mimics infection induced HLH physiology due to disordered cytolysis in response to Lymphocytic Choriomeningitis Virus (LCMV) infection. The FHL model has a more severe phenotype compared to murine TLR9-MAS. FHL mice succumb to disease within 10–21 days, have significantly higher levels of circulating IFN-γ, cytopenia and hepatitis^28^. The combination of high circulating IFN-γ from rapidly proliferating CD8 effector T cells produces tissue damage that in turn results in higher levels of the alarmin IL-33, which exacerbates disease via ST2 receptor in an IFN-γ independent manner^30–32^.

The MAS murine model mimics human disease that is associated with abnormal TLR signaling in patients with Systemic Juvenile Idiopathic Arthritis^29,33,34^. Repeated stimulation of TLR9 produces similar features to MAS with elevated circulating IFN-γ, cytopenia, hepatosplenomegaly and hepatitis^5,28,29,33,35^. As oppose to murine FHL, the MAS model has both a type I and type II interferon response due to TLR9 stimulation resulting in an immune response of both innate and adaptive compartments with a milder contribution of T cells and an overall milder phenotype with mice universally not succumbing to TLR9 repeated stimulation and exhibiting milder features of HLH physiology^28,29,35^. However, prior work has shown that IFN-γ is required for disease progression, whereas Type I interferon is dispensible^29,36^.

We used CXCL9 genetically deficient mice (*cxcl9*^*−/−*^) as well as using AMG487 (Bio-Techne Corp. Minneapolis, MN USA), a pharmacological antagonist of its receptor, CXCR3, to probe the requirement of CXCL9 in both of these murine models. We utilized parameters of liver injury as an example of organ injury associated with FHL and MAS in humans and mice^5,6,9,13,25,29^. Our observations establish that inhibition of CXCL9-CXCR3 interaction does not ameliorate HLH physiology or hepatitis as an example of target organ.

## Results

### CXCL9 deficiency does not alter disease severity in MAS model

**Figure 1:**
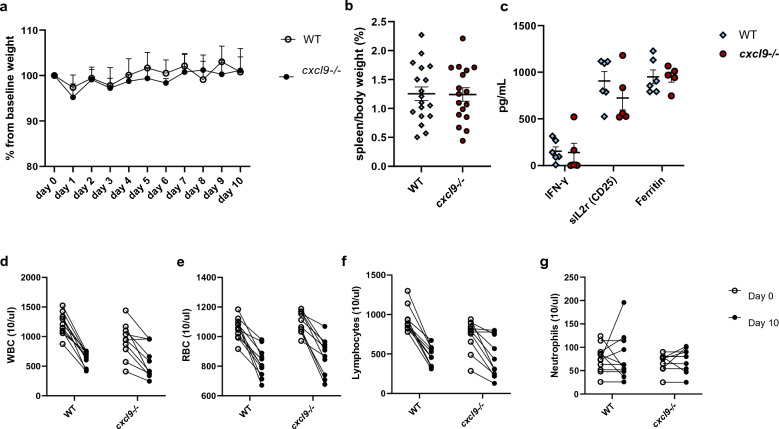
MAS disease parameters in CXCL9 deficient mice. Plots illustrate characteristics of murine TLR9-Induced MAS: (**a**) percent weight change from baseline measured daily, (**b**) spleen size, (**c**) serum cytokines—IFN-γ, sIL2r (CD25) and Ferritin. Complete blood count (CBC) measurements prior to induction (0) and at sacrifice (day 10): (**d**) white blood cell (WBC) count, (**e**) red blood cell (RBC) count, (**f**) lymphocyte count and (**g**) neutrophil count. In (**a**) symbols with error bars mark mean with SEM of daily weights, analyzed with a linear mixed-effects model to allow for missing data due to mouse mortality (n > 17 per group combined 3 experiments). In (**b**,**c**) symbols mark individual mice in each experiment, error bars denote mean with SEM, (**b**,**c**) were analyzed by Student's unpaired 2-tailed *t*-test, p-values in (**d**–**g**) were analyzed using 2-way ANOVA (data combines 3 experiments).

*Cxcl9*^*−/−*^ mice developed MAS comparable to the WT mice in virtually all MAS disease parameters. Groups did not differ in weight change from baseline (Fig. 1a). Both groups developed equivalent splenomegaly (Fig. 1b), elevated serum IFN-γ, soluble IL-2 receptor (sIL2r)/CD25, and Ferritin (Fig. 1c), leukopenia (Fig. 1d), anemia (Fig. 1e) and lymphopenia (Fig. 1f). Neutrophil count was unchanged compared to baseline in both groups (Fig. 1g). There was no difference in 10-day mortality between both groups with no mice succumbing to MAS disease (data not shown). CpG induced splenomegaly and elevated levels of serum IFN-γ were consistent with previously described data when compared to non-MAS mice (Fig. 1c)^5,29,35,37^. sIL2r was equally elevated in both groups when compared to non-MAS mice (mean 515.7 pg/mL, SEM ± 56.62, n = 12, data not shown). Taken together, CXCL9 deficiency does not alter MAS disease parameters.

### CXCL9 deficiency affects intrahepatic T cell and NK cell populations in murine MAS

**Figure 2:**
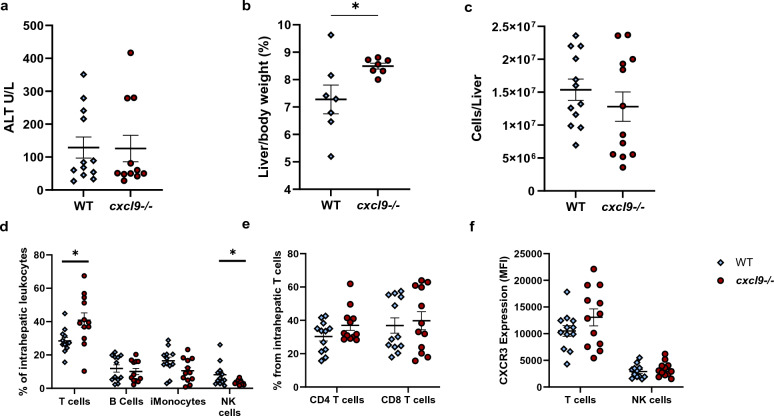
Alterations in intrahepatic inflammatory milieu in CXCL9 deficient MAS. Parameters of liver injury in murine TLR9-Induced MAS: (**a**) ALT and (**b**) liver size. Flow cytometry analysis showing (**c**) total number of intrahepatic leukocytes, (**d**) percent of leukocyte populations, (**e**) percent CD4 and CD8 T cell cells and (**f**) CXCR3 expression by Median fluorescence intensity (MFI) in populations altered by CXCL9 deficiency. Symbols mark individual mice in experiments, all groups were analyzed by Student's unpaired 2-tailed *t*-test, error bars denote mean with SEM. (*p-value < 0.05, **p-value < 0.01).

Investigation of hepatitis parameters showed slight CXCL9-dependant differences in leukocyte infiltration of murine MAS hepatitis. Hepatocellular injury did not differ between WT and *cxcl9*^*−/−*^ groups quantified by Alanine Aminotransferase (ALT) (Fig. 2a). However, *cxcl9*^*−/−*^ mice had significantly larger liver size suggestive of slightly more severe injury (Fig. 2b). Overall hepatic inflammation was not significantly different between groups as measured by total intrahepatic leukocyte count (Fig. 2c). There was great intragroup variability in number of intrahepatic leukocytes and ALT, which could be related to mouse variability in CpG response. Flow cytometric analysis of intrahepatic leukocyte populations revealed a significant, but small increase in T cell and decrease in NK cell percent in the *cxcl9*^*−/−*^ group (Fig. 2d). T Cell subpopulations were not significantly different between groups with a trend in both increases in CD4 and CD8 T cells (Fig. 2e). To investigate if the absence of CXCL9 altered the expression of its receptor, CXCR3, we quantified expression of CXCR3 on T cells and NK cells using Median Fluorescent Intensity (MFI) by flow cytometry. There were no significant changes in T cell or NK cell surface CXCR3 expression (Fig. 2f). In addition, the percent of T and NK cells that were CXCR3+ was not different between groups (data not shown). This suggests that CXCL9-CXCR3 interaction is not the primary driver of lymphocyte recruitment in murine MAS-hepatitis and does not alter hepatocellular injury.

### CXCL9 deficiency does not change disease phenotype in murine FHL

**Figure 3:**
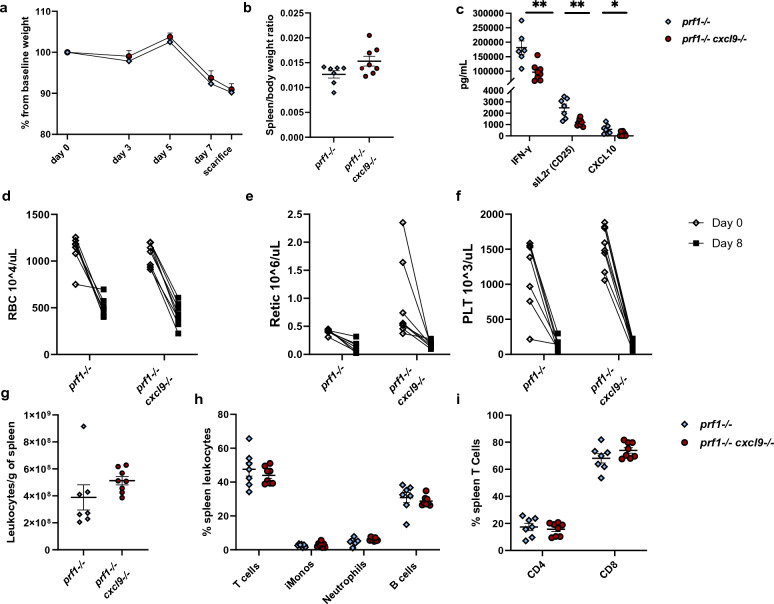
FHL disease phenotype in CXCL9 deficient mice. FHL disease parameters in *prf1*^*−/−*^ and *prf1*^*−/−*^* cxcl9*^*−/−*^ mice including (**a**) percent weight change from baseline measured every 48 h starting day 3 post infection (p.i), (**b**) spleen size, (**c**) serum cytokines- IFN- γ, sIL2r (CD25) and CXCL10. Complete blood count (CBC) measurements on day 0 and at sacrifice (day 8 p.i): (**d**) red blood cell (RBC) count, (**e**) reticulocyte (Retic) count (**f**) platelet (PLT) count. Flow cytometry analysis showing (**g**) total number of spleen leukocytes, (**h**) percent of leukocyte populations, (**i**) percent CD4 and CD8 T cell cells. In (**a**) symbols with error bars mark mean with SEM of daily weights, analyzed with a linear mixed-effects model to allow for missing data due to mouse mortality (n > 7 per group combined 2 experiments). p-values in (**d**–**f**) were analyzed using 2-way ANOVA. In (**b**,**c**,**g**,**h**,**i**) graphs symbols mark individual mice in experiments, all groups were analyzed by Student's unpaired 2-tailed *t*-test, error bars denote mean with SEM. data combines 2 experiments. (*p-value < 0.05, **p-value < 0.01).

To assess the CXCL9 response in FHL model *prf1*^*−/−*^ mice were bred to *cxcl9*^*−/−*^ to produce a *prf1*^*−/−*^* cxcl9*^*−/−*^ strain. Both groups were infected with LCMV to induce FHL and were sacrificed at 8 days post infection when features of disease are present but prior to progressing to high rate of mortality^4^. Similar to human disease, the murine model of FHL produces a much more severe phenotype compared to MAS with severe hypercytokinemia, weight loss/mortality, hepatitis and cytopenias^4,5,25,29,31,32,35,38,39^. Therefore, we hypothesized that the role of CXCL9 in the pathogenesis of *prf1*^*−/−*^ HLH will be more apparent. However, Both *prf1*^*−/−*^ and *prf1*^*−/−*^* cxcl9*^*−/−*^ had no mortality throughout the 8 days post LCMV infection (data not shown) and similar percent weight loss from baseline (Fig. 3a). *Prf1*^*−/−*^* cxcl9*^*−/−*^ group did exhibit a trend toward larger spleen that did not reach statistical significance (Fig. 3b). Evaluation of serum cytokines revealed lower circulating IFN-γ, sIL2r in the *prf1*^*−/−*^* cxcl9*^*−/−*^ group suggesting CXCL9 has a role in lymphocyte activation in FHL as these mediators are produced by the activated T-cells (Fig. 3c)^4,32,40^. Similarly, circulating CXCL10 was lower in the *prf1*^*−/−*^* cxcl9*^*−/−*^ group (Fig. 3c) suggesting that the lack of effect of CXCL9 deficiency on HLH was not due to compensation by CXCL10, and that like IFN-γ and sIL2r, CXCL10 is in part regulated by CXCL9 itself. Disease parameters of marrow dysfunction including circulating red blood cells (RBC), reticulocytes and thrombocytopenia were all similar between *prf1*^*−/−*^ and *prf1*^*−/−*^* cxcl9*^*−/−*^ groups (Fig. 3d–f). Spleen leukocyte quantification by flow cytometry showed higher total leukocyte counts in *prf1*^*−/−*^* cxcl9*^*−/−*^ group compared to *prf1*^*−/−*^ suggestive of increase in retention and consistent with their larger weights, (Fig. 3g) but did not meet statistical significance. The composition of both myeloid and lymphoid populations did not show a difference between groups (Fig. 3h and i). Thus, although CXCL9 may play a role in lymphocyte activation and circulating levels of IFN-γ, the overall FHL phenotype is not affected by its deficiency.

### Murine FHL hepatitis is not ameliorated by CXCL9 deficiency

**Figure 4:**
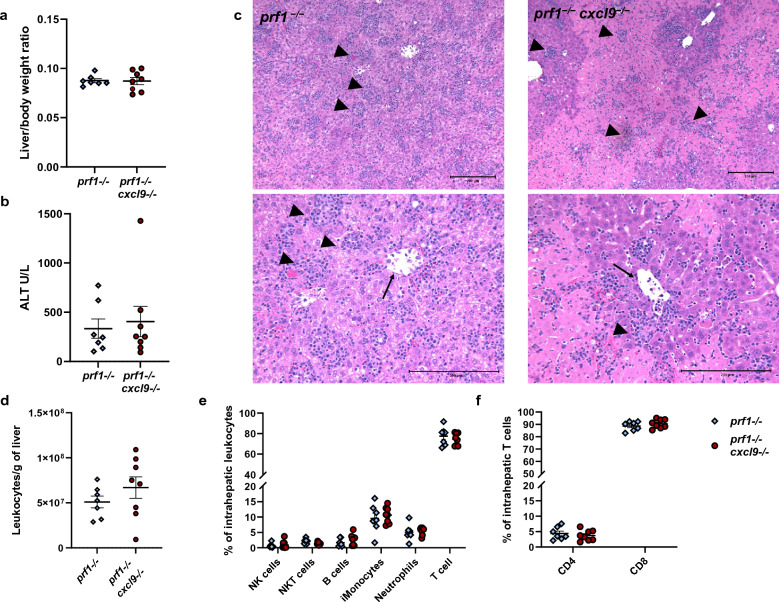
FHL Hepatitis in CXCL9 deficient mice. Parameters of liver injury in murine FHL (**a**) liver size and (**b**) ALT. (**c**) Representative hematoxylin and eosin–stained liver sections from a *prf1*^*−/−*^ mouse (left) and a *prf1*^*−/−*^* cxcl9*^*−/−*^ mouse (right) 8 days after infection. Top panel showing dense lymphocyte lobular infiltrates (arrowheads) in both groups and large area of necrosis *prf1*^*−/−*^* cxcl9*^*−/−*^ mouse in ×10 magnification. Lower panel showing the infiltrates (arrowheads) at higher (20x) magnification as well as features of endothelial injury in both groups including sloughing into lumen (arrows). Flow cytometry analysis showing (**d**) total number of intrahepatic leukocytes, (**e**) percent of leukocyte populations, (**f**) percent CD4 and CD8 T cell cells. Symbols mark individual mice in experiments, all groups were analyzed by Student's unpaired 2-tailed *t*-test, error bars denote mean with SEM (*p-value < 0.05, **p-value < 0.01).

Previous work from our group established that hepatic transcription of *cxcl9* as well as CD8 effector cell and inflammatory monocyte recruitment are dependent on hepatic IFN-γ response^25^. To further investigate the relationship between CXCL9 and leukocyte recruitment in FHL hepatitis, liver injury markers and inflammation in *prf1*^*−/−*^ and *prf1*^*−/−*^* cxcl9*^*−/−*^ groups were analyzed. Liver size was comparable between groups as well as ALT measurement of hepatocellular injury (Fig. 4a,b). Histologically, both groups showed common features of murine FHL hepatitis including dense portal and lobular lymphocytic infiltrates, endothelial injury in central veins and areas of hepatocellular necrosis (Fig. 4c). Analysis of intrahepatic leukocytes by flow cytometry showed a slight trend to increased leukocytes in *prf1*^*−/−*^* cxcl9*^*−/−*^ mice that was not statistically significant (Fig. 4d). There was no difference between groups in percentage of both intrahepatic lymphoid and myeloid cells (Fig. 4e,f). To determine if CXCL9 contributes to organ-infiltrating T cell response, ex vivo stimulation of intrahepatic leukocytes was performed with LCMV peptides (gp33 for CD8 and gp61 for CD4 T cells) or phorbol myristate acetate (PMA)/Ionomycin. There was a decrease in percentage of both CD4 and CD8 IFN-γ+ cells in *prf1*^*−/−*^* cxcl9*^*−/−*^ mice compared to *prf1*^*−/−*^ controls due to PMA/Ionomycin stimulation suggesting a lower total capacity for T-cell IFN-γ response (Supplementary Fig. 1). There was small reduction in CD4 T cells stimulated by gp61 in the *prf1*^*−/−*^* cxcl9*^*−/−*^ group but no difference in IFN-γ+ CD8 T cells stimulated by gp33 (Supplementary Fig. 1). Thus, there may be a small contribution of CXCL9 to intrahepatic T cell IFN-γ production. However, it appears that CXCL9 does not have a significant role in the pathophysiology of murine FHL hepatitis more broadly.

### CXCR3 inhibition does not alter FHL and MAS phenotype

**Figure 5:**
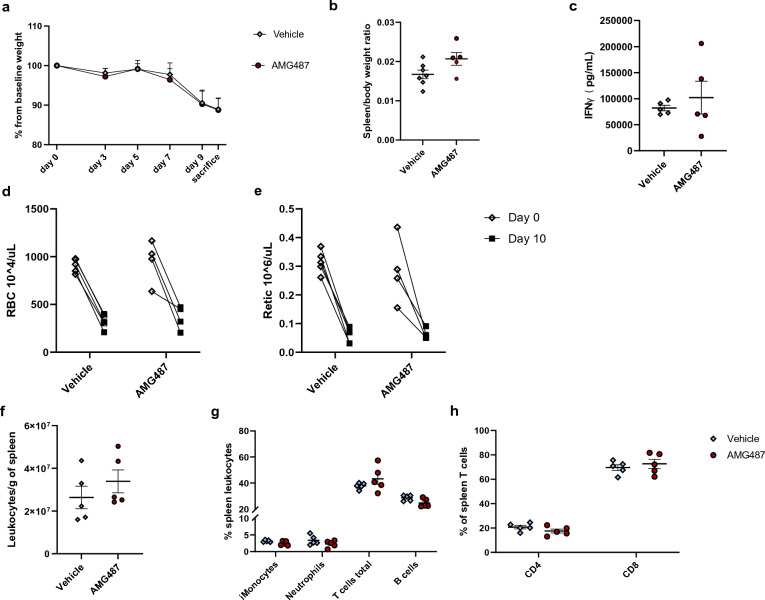
FHL disease phenotype in CXCR3 antagonist (AMG487) treated mice. FHL disease parameters in *prf1*^*−/−*^ mice treated with AMG487 or vehicle including (**a**) percent weight change from baseline measured every 48 h starting day 3 post infection (p.i), (**b**) spleen size, (**c**) serum IFN- γ. Complete blood count (CBC) measurements on day 0 and at sacrifice (day 10 p.i), (**d**) red blood cell (RBC) count and (**e**) reticulocyte (Retic) count. Flow cytometry analysis showing (**f**) total number of spleen leukocytes, (**g**) percent of leukocyte populations, (**h**) percent CD4 and CD8 T cell cells and. In (**a**) symbols with error bars mark mean with SEM of daily weights, analyzed with a linear mixed-effects model to allow for missing data due to mouse mortality (n > 5 per group combined 3 experiments). p-values in (**d**,**e**) were analyzed using 2-way ANOVA. In (**b**,**c**,**f**,**g**,**h**) graphs symbols mark individual mice in experiments, all groups were analyzed by Student's unpaired 2-tailed *t*-test, error bars denote mean with SEM. data combines 2 experiments. (*p-value < 0.05, **p-value < 0.01).

To investigate if the other CXCR3 ligand, CXCL10, or the receptor itself are contributing to murine FHL, we treated *prf1*^*−/−*^ mice infected with LCMV to induce FHL with a CXCR3 antagonist, AMG487, starting at day 3 post infection (p.i). AMG487 is a small molecule inhibitor of CXCR3 that inhibits both lymphocyte and monocyte migration to peripheral tissue in vitro and in vivo^41–47^. We treated mice in the 5mg/kg dosing that was used in other murine experiments that resulted in decreased migration of CXCR3+ cells in vivo^43,46,48^. Because C57BL/6 mice have an innate frame shift mutation within the coding region of *cxcl11* gene resulting in functional deficiency of the chemokine, inhibition of CXCR3 will only reflect CXCL9 and CXCL10 activity in this murine model^49^. There was no difference in weight loss between mice treated with AMG487 vs. Vehicle (Fig. 5a) and overall survival by day 10 was slightly worse in AMG487 group (55%) compared to Vehicle (75%). Spleen size (Fig. 5b) and serum IFN-γ (Fig. 5c) were slightly higher in mice treated with AMG487 but was not statistically significant. Anemia and bone marrow suppression was comparable between both groups (Fig. 5d,e). Similar to mice deficient in CXCL9 (Fig. 3g), the AMG487-treated mice appeared to have no difference in spleen leukocytes compared to controls (Fig. 5f) nor the composition of myeloid and lymphoid cells between the two treatment groups (Fig. 5g,h). increasing the dose of AMG487 (10 mg/kg, 20 mg/kg and 40 mg/kg) did not alter disease phenotype with similar pattern of weight loss, splenomegaly, serum sIL-2r levels and degree of hepatitis (Supplementary Fig. 2). To confirm results from FHL model, WT mice in MAS model were treated with a similar dose of 5 mg/kg AMG487 or vehicle while receiving CpG every 48 h. MAS mice treated with AMG487 did not have a difference in weight loss, and although they did have a larger spleen, they also had a trend towards a decrease in splenocytes (Supplementary Fig. 3). This data suggests that inhibition of CXCR3 did not alter overall disease phenotype in FHL or MAS like results in CXCL9 deficient mice.

### CXCR3 antagonism does not improve FHL and MAS hepatitis

**Figure 6:**
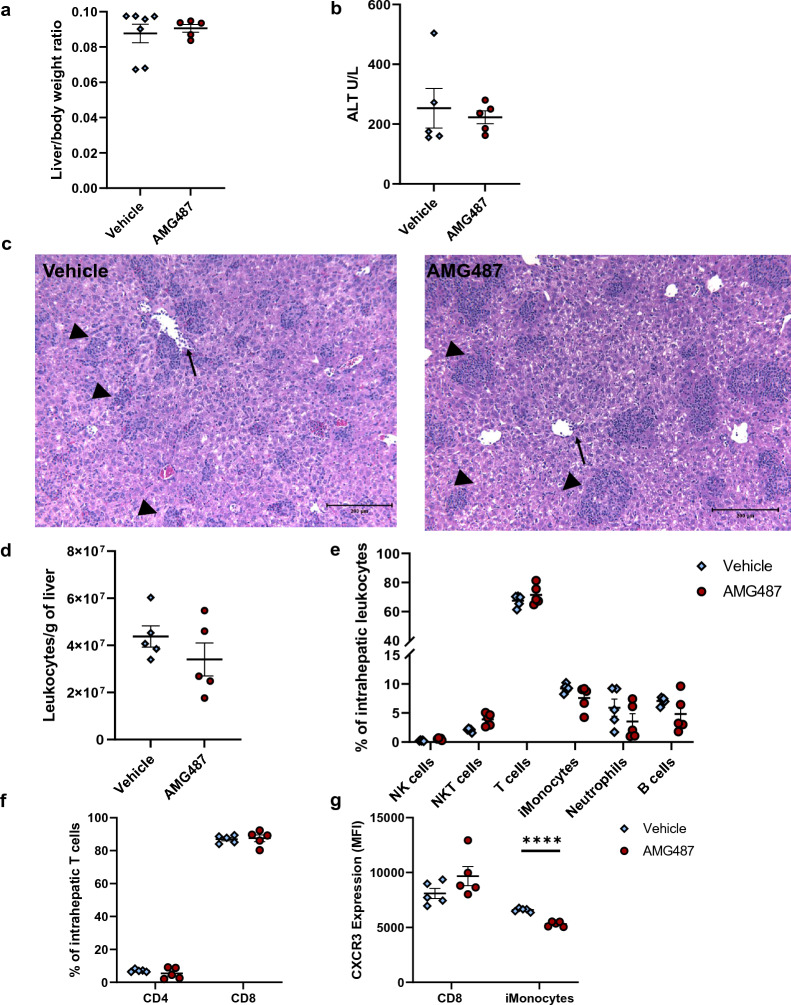
FHL hepatitis in CXCR3 antagonist (AMG487) treated mice. Parameters of liver injury in murine FHL (**a**) liver size and (**b**) ALT. (**c**) Representative hematoxylin and eosin–stained liver sections at ×10 magnification from a *prf1*^*−/−*^ mouse treated with vehicle (left) and a *prf1*^*−/−*^ mouse treated with AMG487 (right) 10 days p.i showing dense lymphocyte lobular infiltrates (arrowheads) as well as features of endothelial injury in both groups including sloughing into lumen (arrows). Flow cytometry analysis showing (**d**) total number of intrahepatic leukocytes, (**e**) percent of leukocyte populations, (**f**) percent CD4 and CD8 T cell cells and (**g**) CXCR3 expression by Median fluorescence intensity (MFI) in populations altered by AMG487 treatment (inflammatory monocytes = iMonocytes). Symbols mark individual mice in experiments, all groups were analyzed by Student's unpaired 2-tailed *t*-test, error bars denote mean with SEM. (*p-value < 0.05, **p-value < 0.01 ***p-value < 0.0001).

Evaluation of quantitative hepatitis parameters including liver size (Fig. 6a) and ALT (Fig. 6b) showed that AMG487 treated mice did not differ compared to those treated with Vehicle. Qualitatively both groups showed comparable histological findings including dense portal and lobular lymphocytic infiltrates, endothelial injury in central veins and areas of hepatocellular necrosis (Fig. 6c). These were comparable to those found in CXCL9 deficient mice (Fig. 4c). Treatment with AMG487 appeared to reduce hepatic leukocytes but was not statistically significant (Fig. 6d). The intrahepatic myeloid and lymphoid populations did not differ significantly (Fig. 6e). In addition, T cells populations were unchanged by treatment of AGM487 (Fig. 6e,f). Lastly, AMG487 did cause a significant inhibition of expression of CXCR3 in intrahepatic inflammatory monocytes (Fig. 6g). This suggests that CXCR3 antagonism does not inhibit tissue recruitment of leukocytes in FHL. Treatment with AMG487 in the MAS model increased the number of intrahepatic leukocytes yet reduced the number of intrahepatic CD8 T-cells and their expression of CXCR3 (Supplementary Fig. 3). Taken together, CXCR3 inhibition by AMG487 had no significant effect on the hepatic inflammatory milieu in murine FHL and MAS. There were minor changes in number of overall intrahepatic leukocytes and expression of CXCR3 in myeloid population in FHL and CD8 T cells in MAS but this therapeutic intervention did not ameliorate liver injury.

## Discussion

Despite the utility of CXCL9 as a diagnostic and a biomarker of IFN-γ activity in primary and secondary HLH, this study revealed that it does not have a significant biological role in pathogenesis of either MAS or FHL murine models. Both genetic deficiency of CXCL9 in TLR9-induced MAS and FHL models as well as pharmacological inhibition of the receptor CXCR3 using AMG487 in FHL model did not ameliorate disease phenotype significantly. Thus, while CXCL9 is a useful marker of IFN-γ activity, it is not the IFN-γ induced effector function contributing to disease in this setting^28^. Previous work from our group showed that hepatic IFN-γ response is critical for development of hepatitis in murine FHL by alteration of hepatic transcription in pathways related to extra cellular matrix production, oxidative stress and leukocyte recruitment and activation^25^. This work contributes insight to the minor contribution of IFN-γ inducible chemokines CXCL9 and CXCL10 through CXCR3 to pathogenesis of disease.

Circulating IFN-γ was reduced in TLR9-idnuced MAS and to a larger degree in FHL when there was deficiency in CXCL9. Previous publications showed in murine models and humans that CXCL9 has an important role in activation of lymphocytes and stimulation of IFN-γ. Rosenblum et al. showed in a murine model of heart allograft rejection that CXCL9 produced by allograft dendritic cells results in skewing toward Th1 response and IFN-γ producing CD8 T cells^50^. Whiting et al. demonstrated that in vitro CXCL9 stimulation of CD4 derived splenocytes results in proliferation and increased number of IFN-γ producing CD4 T cells^51^. Campbell et al. showed that CXCL9 induces IFN-γ production in human PMBC derived from patients with atopic disease^52^. This supports our findings of both decrease in circulating IFN-γ and sIL2r in CXCL9 deficient mice (Figs. 1c and 3c) and reduction of both IFN-γ+ CD8 and CD4 cells stimulated by PMA/Iono and specific LCMV peptide (Supplementary Fig. 3). The findings of elevated circulating IFN-γ and sIL2r were not reproduced by AMG487 inhibition of CXCR3. This suggests either an alternative pathway for CXCL9 mediated lymphocyte activation that is CXCR3 independent or a repressive effect of CXCL10 only seen when CXCL9 is removed. However, CXCL9 deficiency resulted in reduction of circulating CXCL10 as well (Fig. 3c), making the latter less plausible.

CXCR3 antagonism was used in T-cell driven disease models and human trials with varying results^49,53,54^. AMG487 did not ameliorate FHL as a whole or hepatitis specifically in this study. The dosing and frequency in this study was based on 2 studies by Bakheet et al. which showed amelioration of murine arthritis by similar AMG487 dosing regimen^46,48^. Higher doses of AMG487 did not show a change in FHL disease parameters suggesting that our results are not simply due to underdosing (Supplementary Fig. 2). This raises the possibility that treatment with AMG487 may require combination with inhibition T cell activation to reduce both chemotaxis and T cell activation. Miao et al. described that use of AMG487 combined with the calcineurin inhibitor cyclosporine improved murine acute Graft versus Host Disease including in the liver. Mice treated with a combination of AMG487 and cyclosporine had a reduction in intrahepatic T cells, decreased activation of T cells and decreased production of IFN-γ by both CD4 and CD8 T cells compared to AMG487 alone^43^. It is possible that CXCR3 antagonism may also require additional therapy with calcineurin inhibitors to treat FHL/MAS, an area we did not explore. At this juncture it appears AMG487 monotherapy in various dosing does not ameliorate FHL or MAS and may have a deleterious effect with slightly higher mortality in treated mice suggesting this approach may not be a priority for development in humans.

In summary, the current study establishes that despite its utility as a non-invasive biomarker for IFN-γ activity in HLH, CXCL9 does not have a foundational role in pathogenesis of murine models of MAS or FHL. Neither genetic deficiency of CXCL9 nor pharmacological inhibition of its receptor, CXCR3, resulted in amelioration of FHL. The hepatic inflammatory milieu was not altered significantly in MAS and FHL and had no improvement of hepatitis disease parameters. This suggests that IFN-γ driven hepatic injury in FHL is not primarily caused by IFN-γ induced chemokines resulting in T cell and macrophage recruitment but rather by other immune mediated pathways or transcriptional changes in non-immune hepatic constituents that need to be studied further.

## Methods

All methods were performed in accordance with the relevant guidelines and regulations.

### Mice

C57BL/6 (WT), perforin-deficient (C57BL/6-Prf1^tm1Sdz^/J, *prf1*^−/−^), CXCL9-deficient (C57BL/6-*Cxcl9*^*tm1Jmf*^/J, *cxcl9*^*−/−*^) mice were purchased from The Jackson Laboratory and bred in our facility. *prf1*^−/−^ mice were crossed with *cxcl9*^*−/−*^ mice. All Animal studies were performed with approval from Children’s Hospital of Philadelphia Institutional Animal Care and Use Committee and is reported in accordance with ARRIVE guidelines.

### Induction of MAS

MAS was induced by repeated activation of TLR9 as previously described by our group^29^. Briefly, 7–10 week old mice were injected with five doses of 50 µg of CpG1826 intraperitoneally (IP) every 48 h for 9 days. Mice were euthanized at day 10 or if developed significant morbidity or weight loss.

### Induction of FHL

FHL was induced by IP injection of 2 × 10^5^ plaque-forming units of Lymphocytic Choriomeningitis Virus (LCMV) (Armstrong strain). Mice were sacrificed on day 8 in experiments using *prf1*^*−/−*^* cxcl9*^*−/−*^ mice and on day 10 or earlier if developed significant morbidity or weight loss in experiments using AMG487.

### Induction of MAS and FHL and AMG487 treatment

Mice that were *prf1*^*−/−*^ were used in AMG487 experiments received IP injection of 5 mg/kg/dose, or higher in dose discovery experiment, (see Supplementary Fig. 4) AMG487 suspended in DMSO or DMSO only every 48 h starting on day 3 post IP injection with LCMV. In MAS experiments, mice received IP injection of 5 mg/kg/dose AMG487 suspended in DMSO or DMSO only every 48 h in conjunction with CpG 1826 injections to induce MAS.

### Euthanasia and biospecimen collection

Euthanasia was achieved in all experiments by cardiac puncture to collect blood for complete blood count (CBC) with differential and reticulocyte count using Sysmex XT-2000i hematology analyzer at the Children’s Hospital of Philadelphia Translational Core. Alanine aminotransferase (ALT) and aspartate aminotransferase (AST) were measured from serum using a Roche Cobas c311 clinical chemistry analyzer measurement at the Children’s Hospital of Philadelphia Translational Core. Serum IFN-γ, CXCL10 and soluble interleukin-2 receptor (sIL-2r) were measured by enzyme-linked immunosorbent assay (ELISA) using BD Biosciences (IFN-γ) and R&D Systems (CXCL10 and sIL-2r) commercial kits according to protocol. Liver and spleen were resected at time of necroscopy and processed as detailed below.

### Histologic analysis

Unperfused livers were fixed for 24 h in 4% paraformaldehyde and either stained with hematoxylin and eosin (H&E) or embedded in paraffin. Images were acquired on a Leica DM4000B upright imaging scope.

### Flow-cytometric analysis

Intrahepatic leukocytes were isolated using a Percoll (GE Healthcare Life Sciences) density gradient centrifugation. 2/3 of each liver was disrupted using a 70-micron filter. The cell pellet was resuspended in 30% Percoll, layered over 70% Percoll, and centrifuged. The interface formed contained the intrahepatic leukocytes. Splenocytes were isolated by disruption of spleen using a 70-micron filter and then treated with RBC lysis. Cell suspensions from intrahepatic leukocytes and splenocytes were stained with LIVE/DEAD fixable viability dye (Life Technologies) and CD4, CD8α, NK1.1, B220, Ly6C, Ly6G, CD11b, CD44, CD62L, CXCR3 and CD90.2 (BD, Pharmingen, eBioscience and BioLegend). Example of gating strategy can be found in supplemental Figs. 4 and 5. For intracellular cytokine staining Intrahepatic leukocytes (10^6^) were stimulated for 4 h at 37 °C with 2 μg/ml brefeldin A (Sigma), and 2 μM monensin (eBioscience) using 3 conditions: (1) 50 ng/mL phorbol myristate acetate (PMA; Sigma) and 1 µg/mL ionomycin (Cell Signaling Technologies), (2)1.0 μg/mL LCMV gp61 peptide (Anaspec) or (3) 0.2 μg/mL LCMV gp33 peptide (GenScript). After staining with Live/Dead viability dye and surface antigens, cells were stained for IFN-γ (BD Biosceinces) and TNFα (BioLegend) using a Cytofix/Cytoperm kit (BD Bioscineces). All samples were acquired on a CytoFLEX LX flow cytometer (Beckman Coulter, Indiana USA) and analyzed using FlowJo software version 10.8.1.

### Statistical analysis

All data were analyzed in GraphPad Prism 8 using statistical tests indicated in the figure legends and results section. Unless otherwise specified, *P*-values are represented by number or symbols (e.g. *P < 0.05, **P < 0.01, ***P < 0.001 ****P < 0.0001).

## Supplementary Information

Supplementary Figures.

Supplementary Table 1.

## Data Availability

Raw data supporting the conclusion in this manuscript will be made available by the authors, without undue reservation, to any qualified researcher. The corresponding author T.D can be contacted via email (diamondt@chop.edu) and will provide files by written request.
