# Supplementary material for: CXCL9 inhibition does not ameliorate disease in murine models of both primary and secondary hemophagocytic lymphohistiocytosis
Source: Sci Rep. 2023 Jul 29;13:12298. doi: 10.1038/s41598-023-39601-9 (PMC10387083; doi:10.1038/s41598-023-39601-9)
Supplement: Supplementary file 1 — Supplementary Figures. [file 41598_2023_39601_MOESM1_ESM.pptx]

## Slide 1
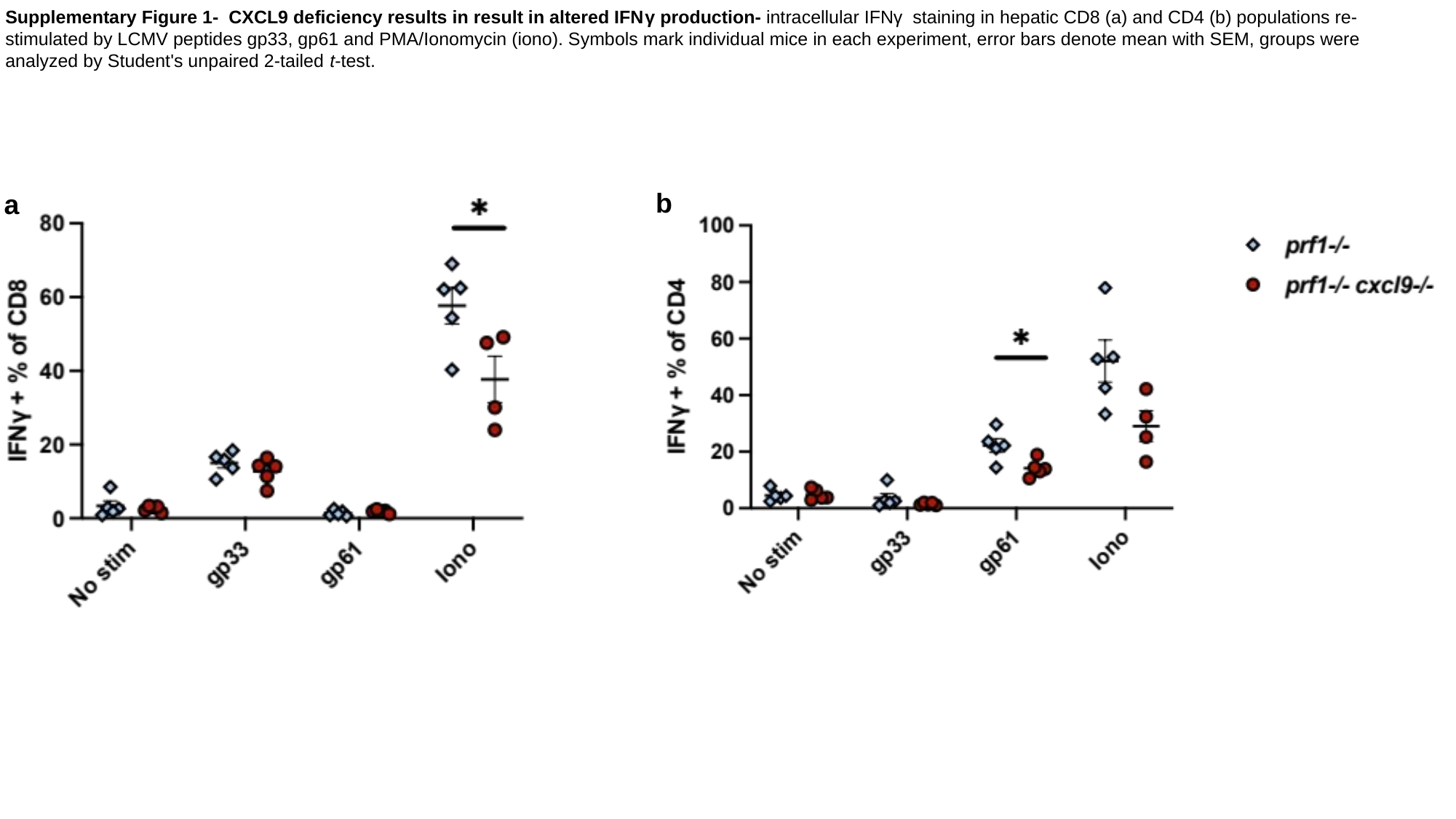

Supplementary Figure 1- CXCL9 deficiency results in result in altered IFNγ production- intracellular IFNγ staining in hepatic CD8 (a) and CD4 (b) populations re-stimulated by LCMV peptides gp33, gp61 and PMA/Ionomycin (iono). Symbols mark individual mice in each experiment, error bars denote mean with SEM, groups were analyzed by Student's unpaired 2-tailed t-test.
b
a

## Slide 2
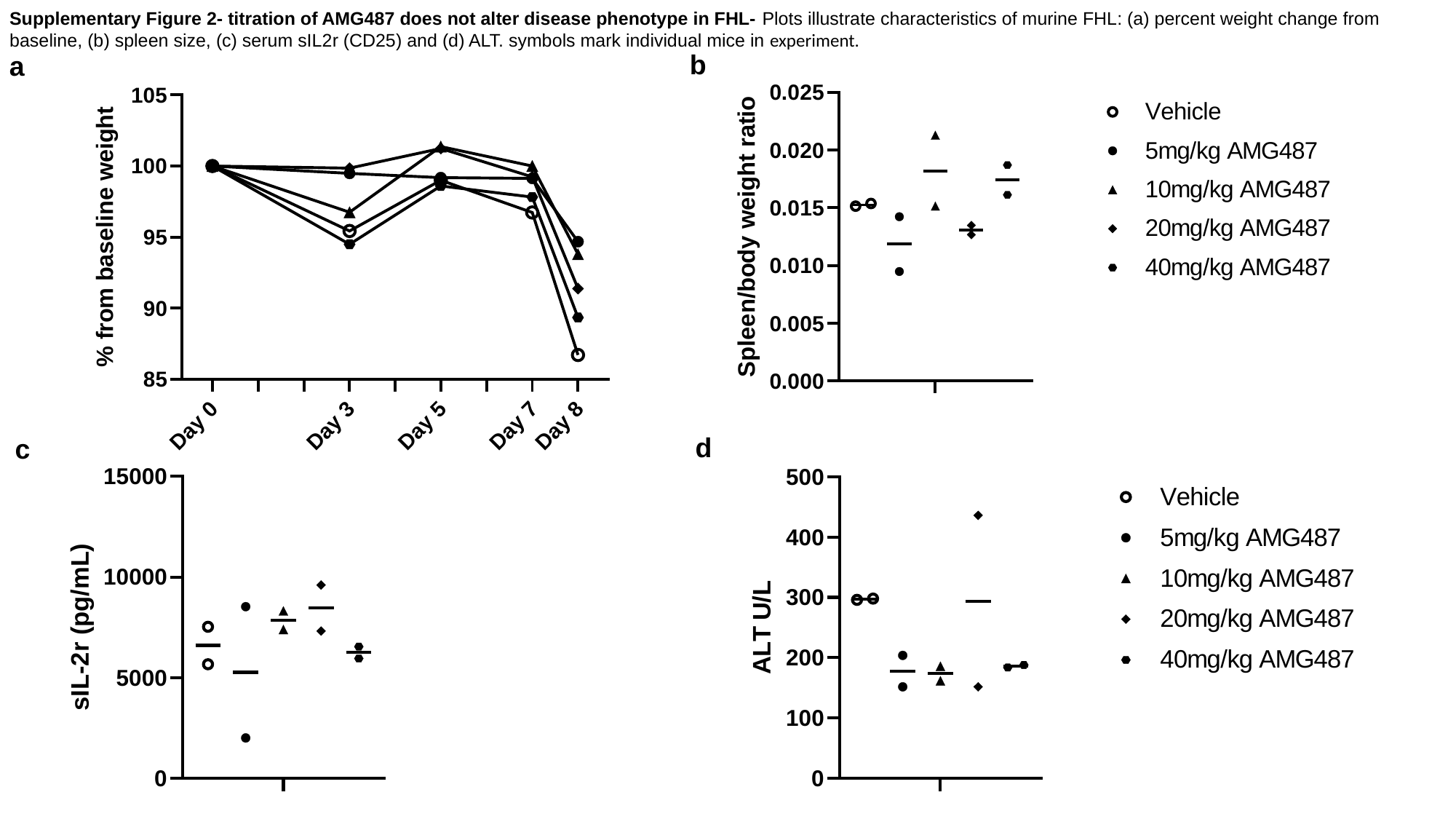

Supplementary Figure 2- titration of AMG487 does not alter disease phenotype in FHL- Plots illustrate characteristics of murine FHL: (a) percent weight change from baseline, (b) spleen size, (c) serum sIL2r (CD25) and (d) ALT. symbols mark individual mice in experiment.
b
a
d
c

## Slide 3
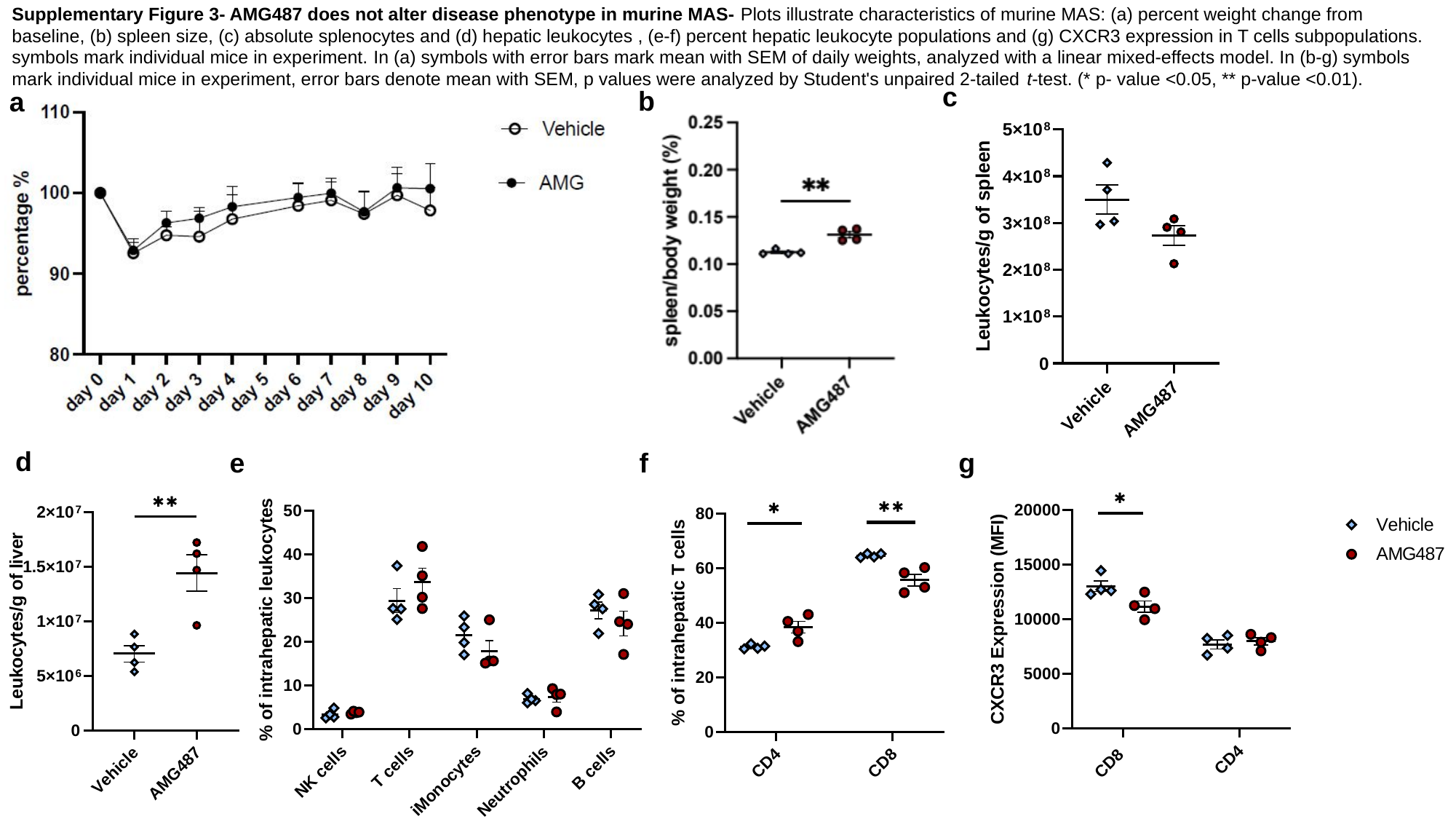

Supplementary Figure 3- AMG487 does not alter disease phenotype in murine MAS- Plots illustrate characteristics of murine MAS: (a) percent weight change from baseline, (b) spleen size, (c) absolute splenocytes and (d) hepatic leukocytes , (e-f) percent hepatic leukocyte populations and (g) CXCR3 expression in T cells subpopulations. symbols mark individual mice in experiment. In (a) symbols with error bars mark mean with SEM of daily weights, analyzed with a linear mixed-effects model. In (b-g) symbols mark individual mice in experiment, error bars denote mean with SEM, p values were analyzed by Student's unpaired 2-tailed t-test. (* p- value <0.05, ** p-value <0.01).
c
b
a
d
e
f
g

## Slide 4
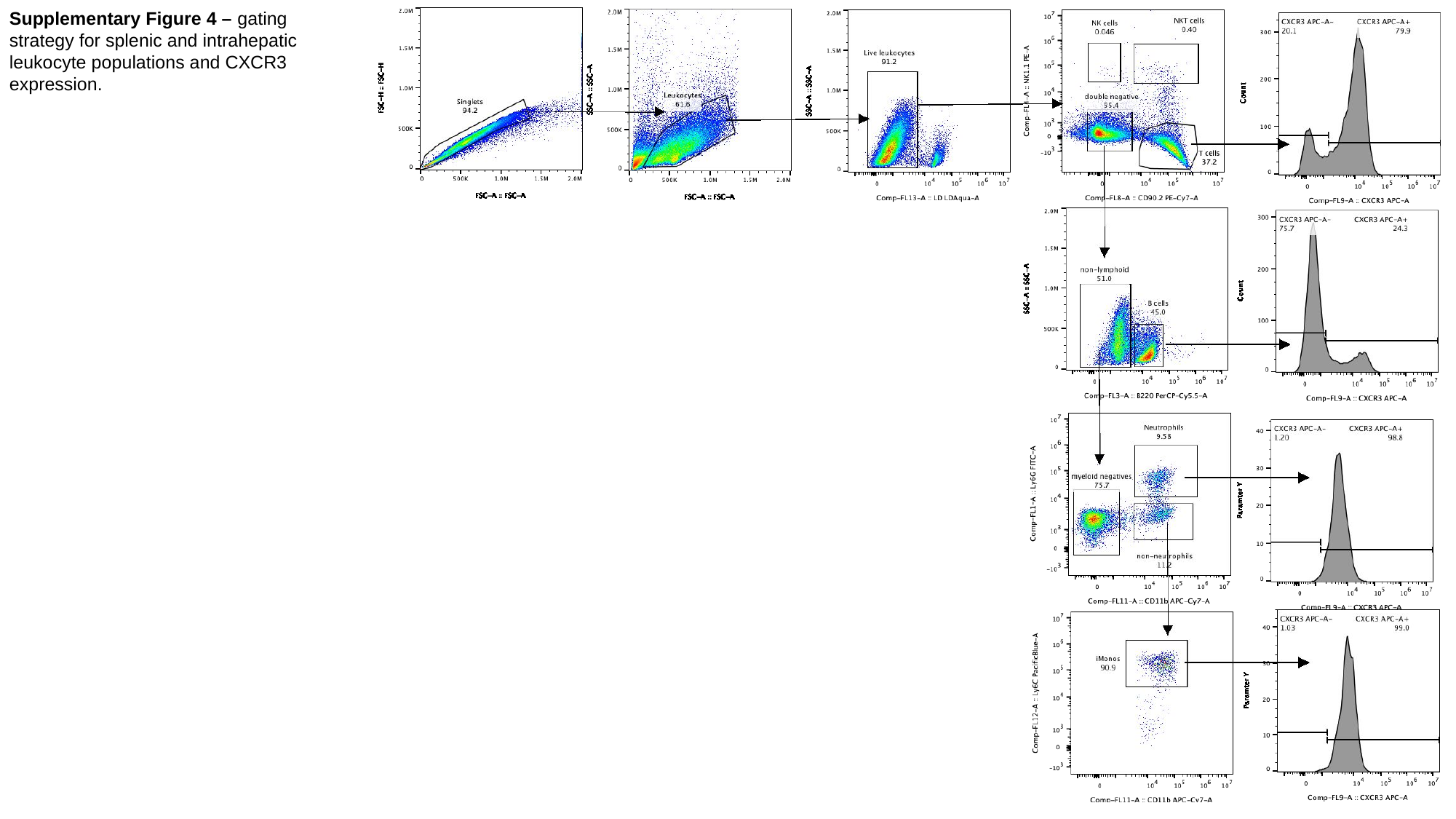

Supplementary Figure 4 – gating strategy for splenic and intrahepatic leukocyte populations and CXCR3 expression.

## Slide 5
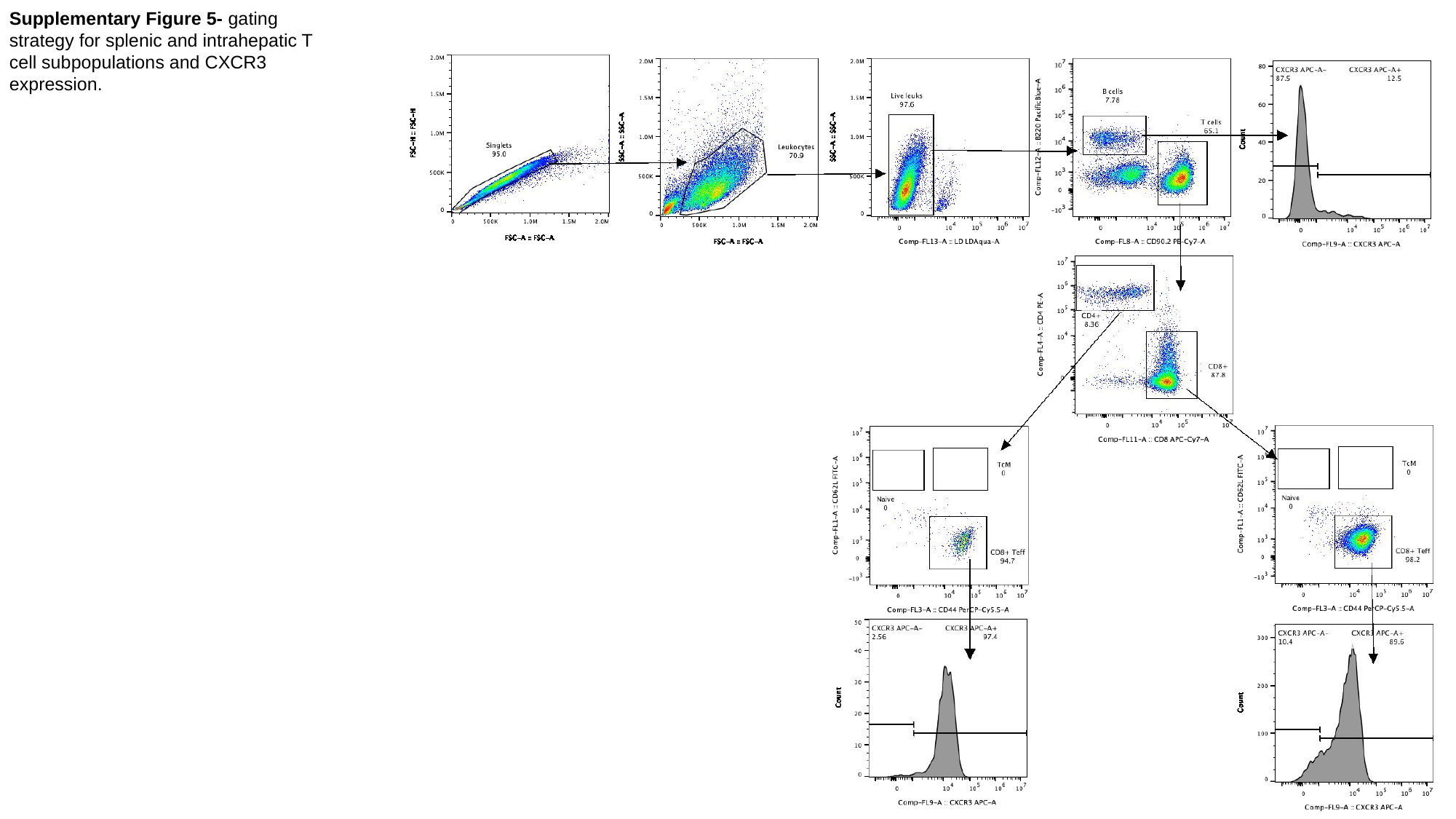

Supplementary Figure 5- gating strategy for splenic and intrahepatic T cell subpopulations and CXCR3 expression.
