# Supplementary material for: CXCL9 inhibition does not ameliorate disease in murine models of both primary and secondary hemophagocytic lymphohistiocytosis
Source: Sci Rep. 2023 Jul 29;13:12298. doi: 10.1038/s41598-023-39601-9 (PMC10387083; doi:10.1038/s41598-023-39601-9)
Supplement: Supplementary file 2 — Supplementary Table 1. [file 41598_2023_39601_MOESM2_ESM.docx]

**Supplementary Table 1- List of Antibodies Used**

| **Fluorophore** | **Antibody** | **Manufacturer** |
| --- | --- | --- |
| Pac Blue | Ly6C | BioLegend |
| Pac Blue | B220 | BioLegend |
| Pac Blue | TNFα | BioLegend |
| Pac Blue | NK1.1 | BioLegend |
| Aqua | LIVE/DEAD | Life Technologies |
| Blue | LIVE/DEAD | Life Technologies |
| FITC | Ly6G | BD Pharmingen |
| FITC | CD44 | BD Pharmingen |
| FITC | CD62L | BioLegend |
| FITC | CD4 | eBioscience |
| FITC | IFN- γ | BioLegend |
| PE | CD4 | BD Pharmingen |
| PE | NK1.1 | BioLegend |
| PerCP-Cy5.5 | CD11b | BD Pharmingen |
| PerCP-Cy5.5 | CD44 | BioLegend |
| PerCP-Cy5.5 | B220 | BioLegend |
| PE-Cy7 | TCRb | BioLegend |
| PE-Cy7 | CD90.2 | BD Pharmingen |
| APC | NK1.1 | BioLegend |
| APC | CD62L | eBioscience |
| APC | CXCR3 | BioLegend |
| APC-Cy7 | CD8α | BioLegend |
| APC-Cy7 | CD11b | BD Pharmingen |
| PE-Texas Red | B220 | BioLegend |
| AF700 | Ly6C | BD Pharmingen |
| BV650 | CD8α | BioLegend |
